# A new species of *Trichopeltis* Pocock, 1894 from southern China, with a checklist and a distribution map of *Trichopeltis* species (Diplopoda, Polydesmida, Cryptodesmidae)

**DOI:** 10.3897/zookeys.725.22014

**Published:** 2017-12-29

**Authors:** Natdanai Likhitrakarn, Sergei I. Golovatch, Ruttapon Srisonchai, Somsak Panha

**Affiliations:** 1 Division of Plant Protection, Faculty of Agricultural Production, Maejo University, Chiang Mai, 50290, Thailand; 2 Institute for Problems of Ecology and Evolution, Russian Academy of Sciences, Leninsky pr. 33, Moscow 119071, Russia; 3 Animal Systematics Research Unit, Department of Biology, Faculty of Science, Chulalongkorn University, Bangkok, 10330, Thailand

**Keywords:** Millipede, *Trichopeltis*, new species, China, Cryptodesmidae

## Abstract

The millipede genus *Trichopeltis* Pocock, 1894 contains 12 described species including a new species from southern China described here. *Trichopeltis sutchariti* **sp. n.** can be distinguished from congeners by its gonopods that are strongly caudolaterally curved and have a prominent, high, curved, densely setose process on each coxa. An updated checklist and a distribution map are provided for all species of the genus.

## Introduction

*Trichopeltis* Pocock, 1894 is the largest genus within the mainly tropical family Cryptodesmidae which currently contains only 12 genera (mostly monotypic) and 39 species (Golovatch 2015, 2016; Golovatch and VandenSpiegel 2017; Liu et al. 2017).

*Trichopeltis* has recently been reviewed and a key provided (Liu et al. 2017). At present, this genus encompasses eleven species that range from the Himalayas of India (Assam and Darjeeling District) and Myanmar to southern China, Laos, Vietnam and Indonesia (Sumatra) (Fig. 5). Five of the species are presumed troglobites: one in Laos, the other four in southern China (Golovatch et al. 2010; Golovatch 2016; Golovatch and VandenSpiegel 2017; Liu et al. 2017). Below the description of one more new congener is presented, the first to be found epigeically in China. A catalogue to all presently known species is also provided, as well as a map showing their distributions.

Since a key to all hitherto known species of *Trichopeltis* is available (Liu et al. 2017), no update is needed.

## Materials and methods

The material was collected from a limestone mountain area in Yunnan, southern China in October 2016. Photographs of live animals were taken in the laboratory using a Nikon 700D digital camera with a Nikon AF-S VR 105 mm macro lens. Specimens were preserved in 75% ethanol, and morphological investigations were carried out in the laboratory with the help of an Olympus SZX7 stereo microscope. Scanning electron micrographs (SEM) were taken with a JEOL, JSM–5410 LV microscope with no metallic coating, and the material returned from stubs to alcohol upon examination. Images of one holotype gonopod were taken in the laboratory and assembled using the “Cell^D^” automontage software of the Olympus Soft Imaging Solution package. The holotype and most of the paratypes are housed in the Museum of Zoology, Chulalongkorn University (**CUMZ**), Bangkok, Thailand. A paratype has also been donated to the collection of the Zoological Museum, State University of Moscow, Russia (**ZMUM**), as indicated in the text.

The collecting site was located by GPS using the WGS84 datum.

In the catalogue sections, D stands for the original description and/or subsequent descriptive notes, K for the appearance in a key, L for the appearance in a species list, R for a new subsequent record, and M for a mere mention.

## Results

### Family Cryptodesmidae Karsch, 1880

#### Genus Trichopeltis

Taxon classificationAnimaliaMicrothyrialesTrichothyriaceae

Pocock, 1894

Trichopeltis Pocock, 1894: 374 (D).
Trichopeltis – Pocock 1895: 789 (K); Attems 1899: 301 (D, K); 1914: 167 (D); 1940: 218 (D, K); Hoffman 1961: 408 (M, K); Golovatch et al. 2010: 71 (D); Golovatch 2015: 156 (M); Golovatch and Akkari 2016: 1 (M); Golovatch and Wesener 2016: 35 (L), Golovatch and VandenSpiegel 2017: 762 (D, M); Liu et al. 2017: 2, 11 (M, D).
Otodesmus Cook 1896: 24 (D). Type species: Trichopeltis watsoni Pocock, 1895), synonymised by Golovatch et al. 2010: 71 (M).
Otodesmus – Attems 1899: 362 (D); Jeekel 1955: 415 (M); Hoffman 1961: 407 (M, K); 1973: 191 (M).

##### Description.

Superficially, a typical genus of Cryptodesmidae, distinguished from other genera in the following combination of characters, the gonopodal ones being the most important.

Body small- to medium-sized (ca 8–21 mm long, ca. 1.7–5.5 mm wide), with 20 segments. Collum flabellate, much broader than head, fully covering it from above; eleven radii at collum’s fore margin dividing it into 12 (sub)equal sectors; dorsal surface tuberculate to areate. Metaterga distinctly tuberculate to areate, usually setose, with at least two irregular transverse rows of tuberculations extending onto paraterga. The latter very short and very wide, subhorizontal, multilobulate at least at caudal and lateral margins. Ozopores highly variable, usually untraceable, when present then barely visible, located near base of paraterga either entirely dorsally or partly dorsally and mainly ventrally, or entirely ventrally. Only coxae 7 or both coxae 6 and 7 distinctly separated to accommodate tips of gonopods. Gonopod aperture usually subcordiform, edges with little or no elevation.

Gonopods ranging from rather simple to relatively complex (Fig. 4), small, usually foliate and held subparallel to each other; telopodites short to rather short, only slightly longer than coxae; the latter usually either bare or poorly setose, more rarely densely setose. Cannula usual, long, slender and falcate, normally not subtended by a median projection of coxa. Prefemoral (setose) part of telopodite making up 1/3–1/2 of the whole; acropodite either distinctly branched (usually with three branches, including an inconspicuous solenomere) or more or less deeply notched apically, seminal groove running entirely on mesal face to end on a more or less distinct caudo-apical solenomere.

##### Type species.

*Cryptodesmus bicolor* Pocock, 1894, by original designation.

##### Other species included.

*T. doriae* Pocock, 1895, *T. feae* Pocock, 1895, *T. watsoni* Pocock, 1895, *T. kometis* (Attems, 1938), *T. latellai* Golovatch, Geoffroy, Mauriès & VandenSpiegel, 2010, *T. cavernicola* Golovatch, 2016, *T. muratovi* Golovatch & VandenSpiegel, 2017, *T. bellus* Liu, Golovatch & Tian, 2017, *T. intricatus* Liu, Golovatch & Tian, 2017 and *T. reflexus* Liu, Golovatch & Tian, 2017.

#### Trichopeltis bicolor

Taxon classificationAnimaliaMicrothyrialesTrichothyriaceae

(Pocock, 1894)

Cryptodesmus bicolor Pocock, 1894: 373 (D).
Trichopeltis bicolor – Pocock 1895: 794 (M); Cook 1896: 25 (M); Attems 1899: 362 (L, K); 1914: 168 (L); 1940: 219 (D, K); Jeekel 1955: 414 (D, R); Hoffman 1961: 407 (M); 1973: 191 (M); Golovatch et al. 2010: 63 (M, K); Golovatch and VandenSpiegel 2017: 762 (L); Liu et al. 2017: 2, 12 (M, K).

##### Records from Indonesia.

West Sumatra, Singkarah, 1,800 m a.s.l. (Pocock 1894; Jeekel 1955); Anai Cleft, 500 m a.s.l. (Jeekel 1955).

#### Trichopeltis doriae

Taxon classificationAnimaliaMicrothyrialesTrichothyriaceae

Pocock, 1895

Trichopeltis doriae Pocock, 1895: 792 (D).
Trichopeltis Doriae – Attems 1899: 362 (L, K); 1914: 168 (L).
Trichopeltis doriae – Attems 1936: 244 (R); 1940: 221 (D, K); Jeekel 1955: 415 (M); Hoffman 1973: 191 (M); Golovatch et al. 2010: 63 (M, K); Golovatch and VandenSpiegel 2017: 762 (L); Liu et al. 2017: 2, 12 (M, K).

##### Record from Myanmar.

Yado, Carin Asciuii Cheba, 1,200–1,300 m a.s.l. (Pocock 1895; Attems 1936).

#### Trichopeltis feae

Taxon classificationAnimaliaMicrothyrialesTrichothyriaceae

Pocock, 1895

Trichopeltis feae Pocock, 1895: 793 (D).
Trichopeltis Feae – Attems 1899: 362 (L, K); 1914: 168 (L).
Trichopeltis feae – Attems 1936: 244 (R); 1940: 220 (D, K); Jeekel 1955: 415 (M); 1965; 124 (L); Hoffman 1973: 191 (M); Golovatch et al. 2010: 63 (M, K); Golovatch and VandenSpiegel 2017: 762 (L); Liu et al. 2017: 2, 12 (M, K).

##### Records from Myanmar.

Village of Chiala, Carin Asciuii Ghecù, 1,200–1,600 m (Pocock 1895); Between Namkham and Kwangmu, 2,500 feet; Mule track between Hosi and Mio-Hsao, 3,700–4,400 feet; and North Shan States (Attems 1936).

#### Trichopeltis watsoni

Taxon classificationAnimaliaMicrothyrialesTrichothyriaceae

Pocock, 1895

Trichopeltis watsoni Pocock, 1895: 793 (D).
Trichopeltis watsoni – Cook 1896: 24 (M); Attems 1936: 244 (R); 1940: 219 (D, K); Jeekel 1955: 415 (M); Hoffman 1961: 408 (M); 1973: 191 (M); Golovatch et al. 2010: 63 (M, K); Shelley 2014: 5 (M); Golovatch and Wesener 2016: 35 (L); Golovatch and VandenSpiegel 2017: 762 (L); Liu et al. 2017: 2, 12 (M, K).
Otodesmus watsoni – Cook 1896: 25 (M); Attems 1899: 362 (L); Hoffman 1961: 408 (M).
Trichopeltis Watsoni – Attems 1899: 362 (L, K); 1914: 68 (L).
Trichodesmus (sic!) watsoni – Attems 1936: 244 (R); Rajulu 1970: 136 (L).

##### Records from Myanmar.

Chin Hill (upper Burma) (Pocock 1895) and Waseru Choung River, south boundary of North Arakarn; India: Assam, Gauhati (Guwahati); Samagutung; Mangaldai district; Eastern Himalayas, Darjeeling district, Pashok; Bengal, Howrah opposite Calcutta (= Kolkata); Bangladesh: Chittagong Hills Tracts, Shishgk Valley (Attems 1936).

#### Trichopeltis kometis

Taxon classificationAnimaliaMicrothyrialesTrichothyriaceae

(Attems, 1938)

Niponielle (sic!) kometis Attems, 1938: 244 (D).
Onomatoplanus kometis – Attems 1940: 222 (D); 1953: 179 (R).
Pseudoniponiella kometis – Verhoeff 1942: 431 (D); Golovatch 2015: 156 (M).
Niponia kometis – Golovatch 1983: 180 (L); Enghoff et al. 2004: 41 (L); Likhitrakarn et al. 2014: 479 (L); 2015: 181 (L).
Trichopeltis deharvengi Golovatch et al., 2010: 64 (D); Golovatch 2015: 156 (M).
Trichopeltis kometis – Golovatch and Akkari 2016: 2 (D); Golovatch 2016: 33 (M), Golovatch and VandenSpiegel 2017: 757 (L); Liu et al. 2017: 2, 12 (M, K).

##### Records from Vietnam.

Khanhhoa Province, Mt Hon Ba (Nhatrang); Lam Dong Province, Mt Diling, 1,000 m a.s.l. (S. Annam); Dalat (S. Annam), 1,500 m a.s.l.; Dalat, Camli, 1,500 m a.s.l.; Peak Langbian; Danang Province, Mt Bana, 1,400 m a.s.l. (Attems 1938); Lamdong Province, Peak Langbian; Dalat; Danang Province, Mt Bana, 1,500 m a.s.l.; Laocai Province, Mt. Fanxipan; Laichau Province, Laichau (Attems 1953); Lam Dong Province, Dalat, Peak Lang Bian, below summit (ca 2,030 m a.s.l.), (Golovatch et al. 2010); Laos: Xiang Khouang Province, Xieng Khouang; Luang Prabang Province, Luang Prabang; Sainyabuli Province, Paklay; Champasak Province, Bolaven Plateau; Cambodia: Kratie Province, Kratie (Mekong) (Attems 1953).

#### Trichopeltis latellai

Taxon classificationAnimaliaMicrothyrialesTrichothyriaceae

Golovatch, Geoffroy, Mauriès & VandenSpiegel, 2010

Trichopeltis latellai Golovatch et al., 2010: 66 (D).
Trichopeltis latellai – Golovatch and VandenSpiegel 2017: 762 (L); Liu et al. 2017: 2, 12 (M, K).

##### Records from China.

Guizhou Province, Qianxi County, Hong Lin Town, Cave Chang Tu Dong; Cave Tiao Shuz Dong (Golovatch et al. 2010).

#### Trichopeltis cavernicola

Taxon classificationAnimaliaMicrothyrialesTrichothyriaceae

Golovatch, 2016

Trichopeltis cavernicola Golovatch, 2016: 34 (D).
Trichopeltis cavernicola – Golovatch and VandenSpiegel 2017: 765 (D, R); Liu et al. 2017: 2, 12 (M, K).

##### Records from Laos.

Khammouane Province, Ban Naden, Cave Tham Namlat, 17.504969°N, 105.385598°E, ca. 180 m a.s.l. (Golovatch 2016) and ca. 65 km north of Thakaek, Cave Tham Nam Lod (Golovatch & VandenSpiegel, 2017).

#### Trichopeltis muratovi

Taxon classificationAnimaliaMicrothyrialesTrichothyriaceae

Golovatch & VandenSpiegel, 2017

Trichopeltis muratovi Golovatch & VandenSpiegel, 2017: 757 (D).
Trichopeltis muratovi – Liu et al. 2017: 2, 12 (M, K).

##### Record from Laos.

Xieng Khoung Province, ca. 9 km northwest of Vieng Thong, secondary tropical forest, 20°08.466'N, 103°20.099'E, ca. 870–910 m a.s.l. (Golovatch and VandenSpiegel 2017).

#### Trichopeltis bellus

Taxon classificationAnimaliaMicrothyrialesTrichothyriaceae

Liu, Golovatch & Tian, 2017

Trichopeltis bellus Liu et al., 2017: 2, 12 (D).

##### Record from China.

Yunnan Province, Qujing City, Luoping County, Machang village, Cave Shuiyuan Dong, 24°49'33"N, 104°21'48"E, 1,530 m a.s.l. (Liu et al. 2017).

#### Trichopeltis intricatus

Taxon classificationAnimaliaMicrothyrialesTrichothyriaceae

Liu, Golovatch & Tian, 2017

Trichopeltis intricatus Liu et al., 2017: 5, 12 (D).

##### Record from China.

Yunnan Province, Kunming City, Shilin County, Guishan Town, Cave Haiyi I Dong, 24°38'50"N, 103°32'49"E, 1,890 m a.s.l. (Liu et al. 2017).

#### Trichopeltis reflexus

Taxon classificationAnimaliaMicrothyrialesTrichothyriaceae

Liu, Golovatch & Tian, 2017

Trichopeltis reflexus Liu et al., 2017: 8, 12 (D).

##### Record from China.

Hunan Province, Chenzhou City, Linwu County, Xianghualing Town, Cave II Dong (Liu et al. 2017).

#### Trichopeltis sutchariti sp. n.

Taxon classificationAnimaliaMicrothyrialesTrichothyriaceae

http://zoobank.org/99801C98-30DD-4939-B346-4D279B80273F

**Figure 1.**
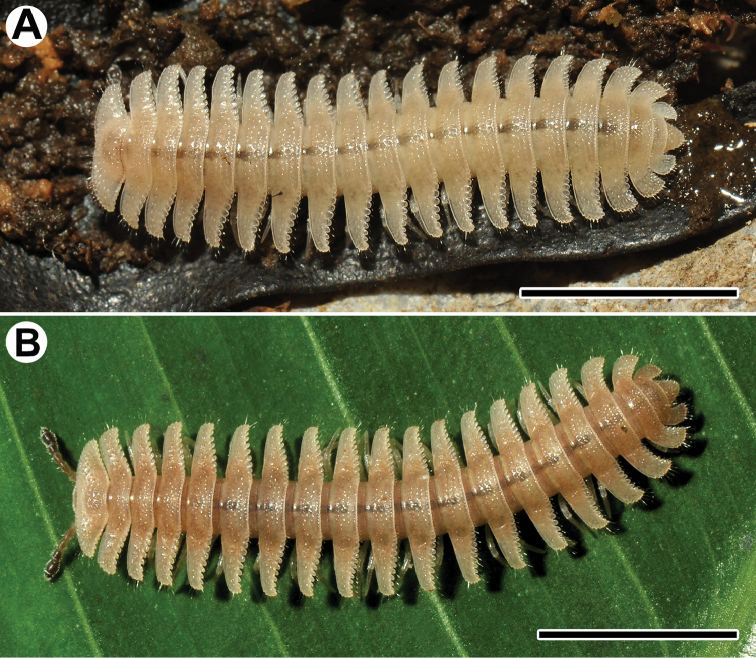
Habitus, live colouration of *Trichopeltis sutchariti* sp. n., **A** ♀ paratype **B** ♂ holotype. Scale bar: 5 mm.

**Figure 2.**
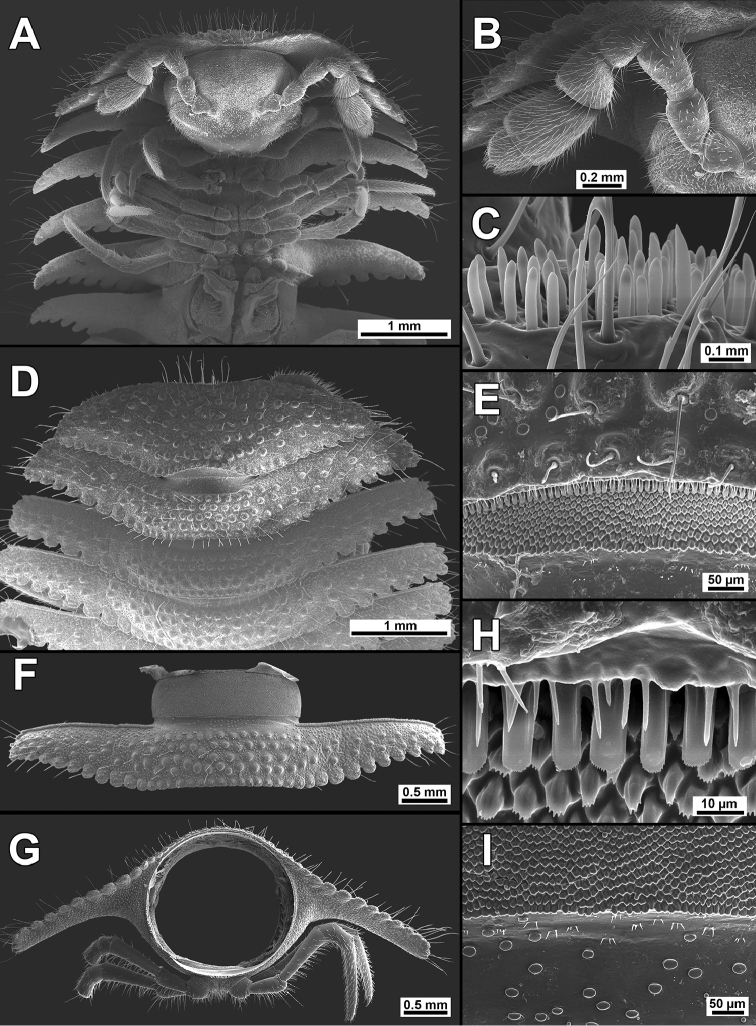
*Trichopeltis sutchariti* sp. n., ♂ paratype. **A, D** anterior part of body, ventral and dorsal views, respectively **B** antenna, ventral view **C** bacilliform sensilla on antennomere 5, lateral view **E** prozona of segment 2, dorsal view **H** limbus of collum, dorsal view **F** midbody segment, dorsal view **G** cross-section of a midbody segment, caudal view **I** enlarged prozona and stricture between pro- and metazona of a midbody segment, dorsal view.

**Figure 3.**
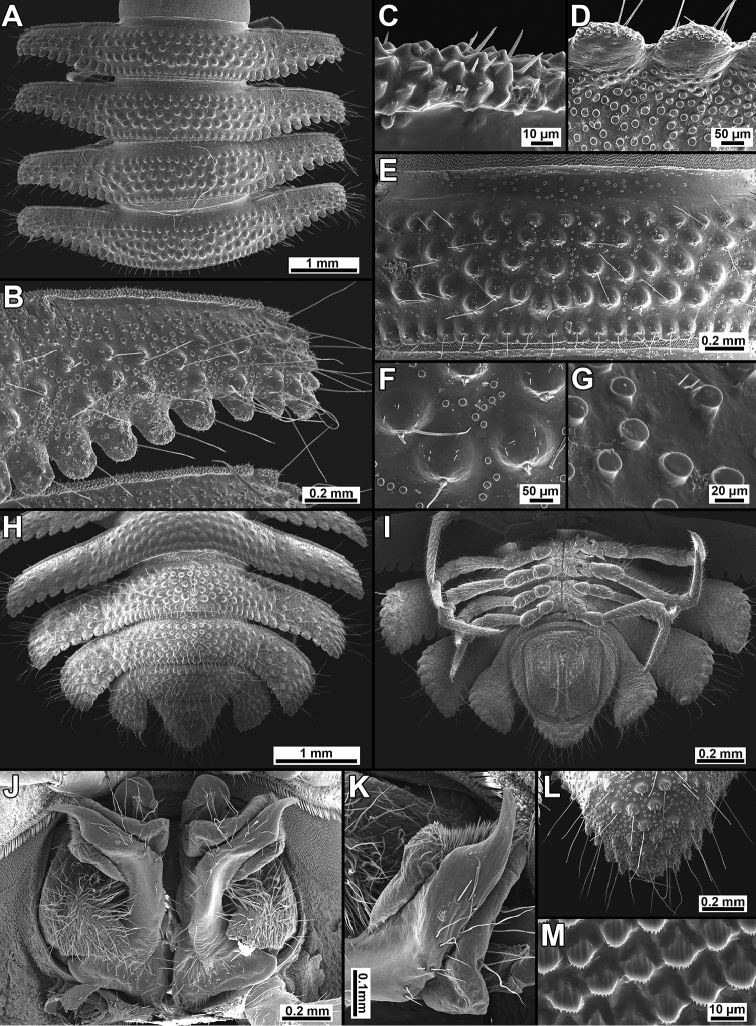
*Trichopeltis sutchariti* sp. n., ♂ paratype. **A** segments 9–12, dorsal view **B** paraterga of segment 10, dorsal view **C** anterior edge of paraterga, dorsal view **D** paraterga, lateral view **E** metaterga of segment 9, dorsal view **F** setigerous tuberculations on metaterga, dorsal view **G** spherical knobs on metaterga, dorsal view **H, I** posterior part of body, dorsal and ventral views, respectively **J, K** gonopod, ventral and sublateral views, respectively **L** enlarged epiproct, dorsal view **M** enlarged prozona, dorsal view. SEM without metallic coating.

**Figure 4.**
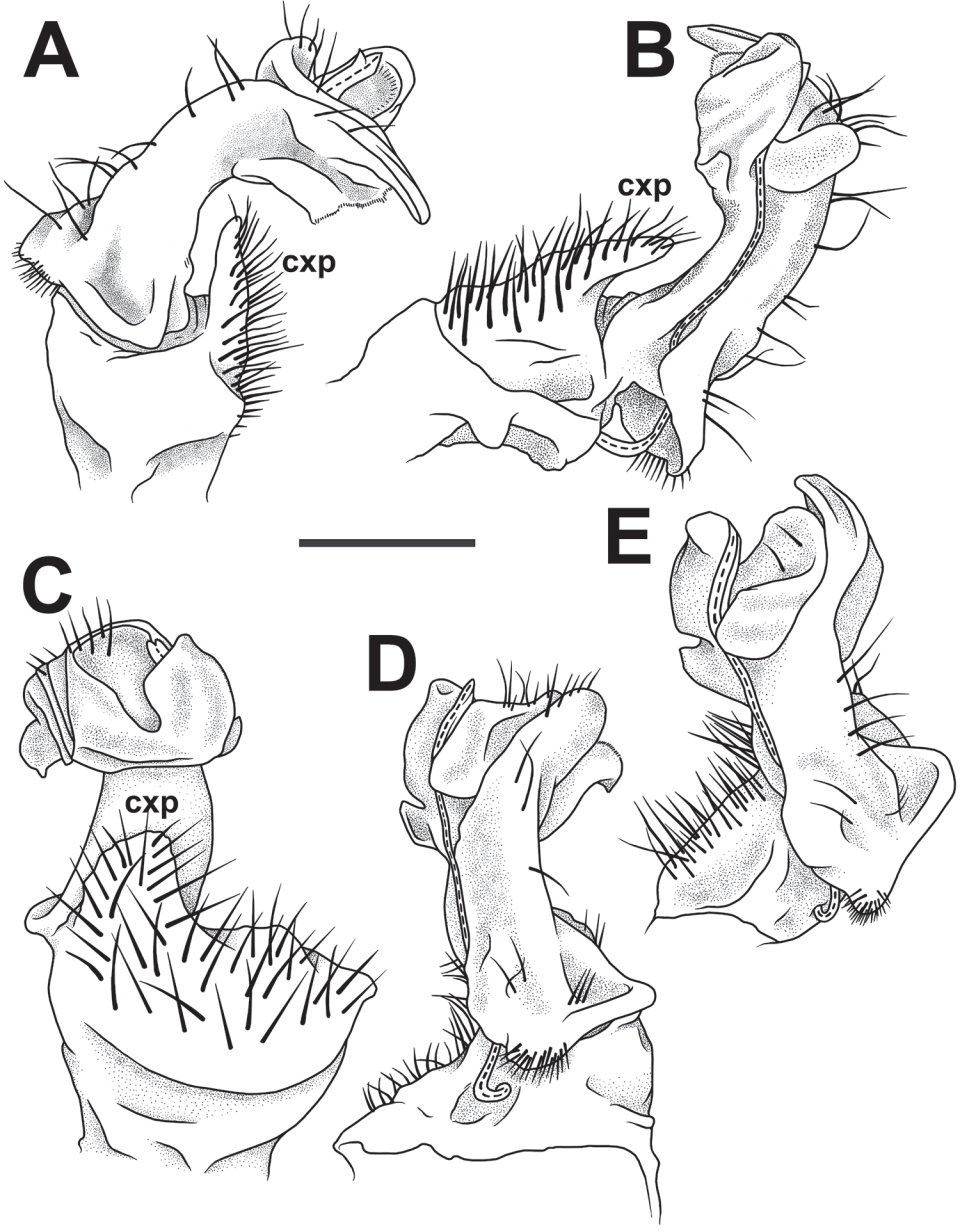
*Trichopeltis sutchariti* sp. n., ♂ holotype. **A–E** right gonopod, subfrontal, mesal (mirror image), sublateral, subcaudal and subfrontal views, respectively. Scale bar: 0.4 mm. Del. N. Likhitrakarn.

**Figure 5.**
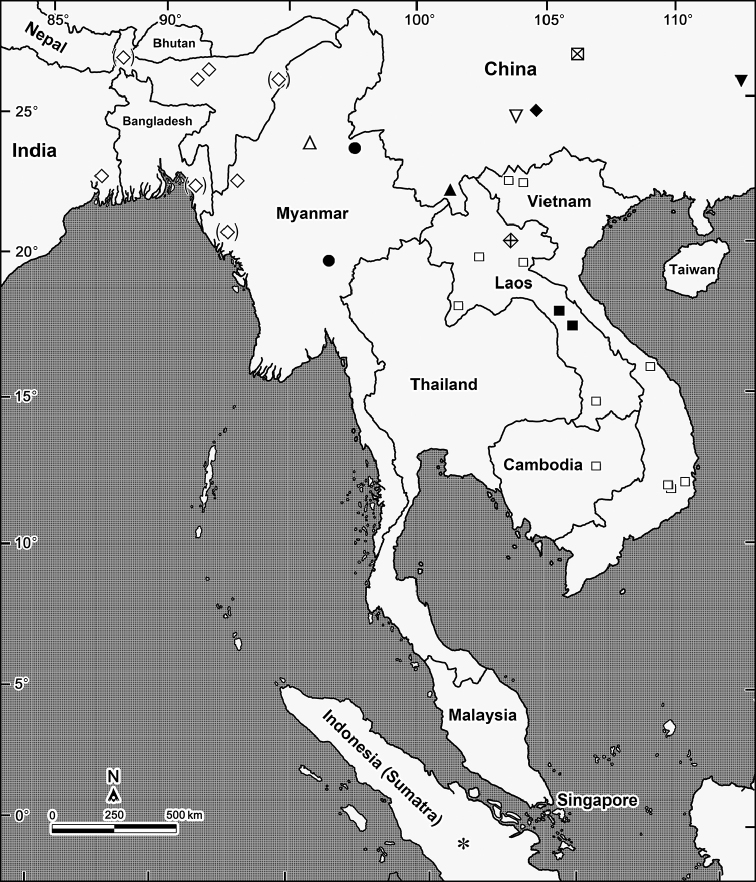
Distribution of 12 currently known species of *Trichopeltis*. Open diamonds: *T. watsoni* Pocock, 1895; crossed open square: *T. latellai* Golovatch, Geoffroy, Mauirès & VandenSpiegel, 2010; open triangle: *T. doriae* Pocock, 1895; filled circle: *T. feae* Pocock, 1895; inverted open triangle: *T. intricatus* Liu, Golovatch & Tian, 2017; filled diamond: *T. bellus* Liu, Golovatch & Tian, 2017; inverted filled triangle: *T. reflexus* Liu, Golovatch & Tian, 2017; filled triangle: *T. sutchariti* sp. n.; crossed open diamond: *T. muratovi* Golovatch & VandenSpiegel, 2017; filled squares: *T. cavernicola* Golovatch, 2016; open squares: *T. kometis* (Attems, 1938); asterisk: *T. bicolor* (Pocock, 1984).

Figs 1 , 2 , 3 , 4 , 5

##### Type material.

Holotype ♂ (CUMZ), China, Yunnan Province, Xishuangbanna County, Mengla, 213 National Road, near Menglunzhen, Munlun village, 578 m a.s.l., 21°56'40"N, 101°13'45"E, 25.10.2016, leg. J. Sutcharit & S. Panha.

##### Paratypes.

2 ♂♂, 1 ♀ (CUMZ), 1 ♀ (ZMUM), same locality, together with holotype.

##### Name.

Honours Jirasak Sutcharit (CUMZ), one of the collectors.

##### Differential dagnosis.

This new species seems to be particularly similar to *T. bellus* Liu, Golovatch & Tian, 2017 and *T. intricatus* Liu, Golovatch & Tian, 2017, both from caves in southern China (Liu et al. 2017), since they all are distinguished by the presence of abundant long setae on the lateral face of the gonopodal coxae, and by highly complex gonopodal telopodites. However, *T. sutchariti* sp. n. differs from them, as well from all other congeners in that its gonopodal telopodites are noticeably curved caudolaterad, and there is a strong, curved, laterally densely setose process (**cxp**) on each of the gonopodal coxae (Fig. 4).

##### Description.

Length 13.9–15.2 (♂) or 14.2–14.5 mm (♀), width of midbody pro- and metazona 1.7–1.9 and 4.8–5.1 mm (♂) or 1.8–2.0 and 4.4–5.2 mm (♀), respectively.

*Colouration* of live animals uniformly whitish yellow (Fig. 1A), sometimes light red-brownish mid-dorsally (Fig. 1B); head, legs and venter whitish yellow to pallid, antennae light brown, increasingly infuscate distally; colouration in alcohol, after eight months of preservation, entirely pallid, only antennae still infuscate (brown) distally.

*Clypeolabral region* and vertex densely pilose, epicranial suture distinct. Antennae rather short and clavate (Figs 1B, 2A, B), reaching segment 3 (♂, ♀) when stretched laterally or ventrolaterally; in length, antennomere 6 > 3 > (4 = 2) > 5; antennomeres 5–7 each with a compact apicodorsal group of bacilliform sensilla (Fig. 2C). Body with 20 segments (Fig. 1A, B), composed of collum plus 17 podous and one apodous ring, plus telson. In width, head << collum < segment 2 <3 < 4 <5–17; thereafter body rapidly tapering towards telson (Fig. 3H).

*Tegument* dull, prozonae finely shagreened (Figs 2E, I, 3M); metaterga densely tuberculate and setose (Figs 2D, E, F, 3A, B, E, H). Fore and caudolateral margins of collum, as well as anterolateral, lateral and caudal margins of following paraterga evidently crenulate-lobulate, these lobulations being slightly larger at caudal margins of paraterga (Figs 2D, 3A, H).

*Collum* completely covering the head from above, regularly convex at fore margin, concave caudally, tuberculations arranged in 8–9 irregular transverse rows of evident setigerous knobs with abundant spherical granulations (Fig. 2D); caudal corner of paraterga narrowly rounded, declined ventrad, but not projecting beyond rear tergal margin (Fig. 2A).

*Dorsum* convex, postcollum paraterga flat, very broad and long, narrowly rounded laterally, evidently and regularly declivous and continuing the outline of dorsum; anterior edge straight, rib-shaped, forming a distinct shoulder, abundantly microgranulate and micropilose (Fig. 3C); tips of paraterga reaching level of venter, directed increasingly caudolaterad starting with segment 14, drawn behind rear tergal margin on segments 16–18 and strongly curved caudad on segment 19 (Fig. 3H); metatergal tuberculations arranged in 5–6 irregular transverse rows of small, round, setigerous knobs surrounded with abundant spherical granulations, these partly extending onto paraterga (Figs 2E, I, 3E, F, G); tergal setae rather short, slender and simple, mostly retained in caudal row on each metatergum (Fig. 2E), macrochaetae absent.

*Limbus* a row of simple, relatively short, tongue-shaped protuberances, abundantly microdenticulate apically (Fig. 2E, H). Ozopores invisible, pore formula untraceable. Pleurosternal carinae with complete crests only on segments 2 and 3 (♀) or with a sharp caudal tooth on segments 2 and 3, but segment 4 with a small rounded denticle (♂), thereafter absent.

*Axial line* absent. Stricture dividing pro- and metazonae broad, shallow and smooth, with abundant spherical granulations dorsally and microgranulate laterally (Fig. 3E).

*Epiproct* (Fig. 3H, L) conical, flattened dorsoventrally, microtuberculate, with four strong apical papillae. Hypoproct roundly subtrapeziform (Fig. 3I), 1+1 caudal setae clearly separated, borne on small, but evident knobs.

*Sterna* usual, sparsely setose, without modifications, cross-impressions evident (Fig. 3I).

*Legs* very long and slender, without modifications (Figs 2G, 3I), ca. 1.3–1.4 times as long as paratergal width (♂, ♀) (Figs 1–3); in length, femora = tarsi >> prefemora > tibiae > coxae and postfemora (Fig. 2G); gonapophyses on ♂ coxae 2 small cones; neither adenostyles nor tarsal brushes present. Gonopod aperture transversely ovoid, caudal and lateral margins thin, only slightly elevated (Fig. 2A).

*Gonopods* (Figs 2A, 3J, K, 4) complex. Each coxa with a conspicuous, high, curved, laterally densely setose process (cxp). Telopodite stout, clearly curved caudolaterad (Fig. 3J), approximately twice as long as coxal process, divided by a notch, but compact apically (Fig. 4).

##### Remarks.

All five specimens were taken from a rather large population found on limestone rocks, as well as on tree trunks during the rainy season. It seems noteworthy that the surface structures illustrated in the new species, such as the sculpture of the prozonae and the shape of the limbus (Fig. 2H, I), perfectly match the findings of Akkari and Enghoff (2011) in other genera/species of the same family.

## Conclusions

At present, the genus *Trichopeltis* comprises 12 species ranging from the Himalayas of India (one species), through Bangladesh (one species), Myanmar (three species), to China (five species), Laos (three species), Vietnam, Cambodia and Indonesia (one species each) (Fig. 5).

Most of the species seem to be highly localised endemics, this being especially true of the five presumed troglobionts. There are only three congeners, all epigean, which are relatively widespread: *T. kometis*, found in Vietnam, Laos and Cambodia; *T. feae*, recorded from several localities in Myanmar; and *T. watsoni*, reported from Mynamar, Bangladesh and the Himalayas of India (Fig. 5).

Such a distribution pattern of *Trichopeltis* clearly suggests its Indo-Malayan (= Oriental) roots and there is little doubt that more new and interesting species will be discovered and additional localities recorded in future.

## Supplementary Material

XML Treatment for Trichopeltis

XML Treatment for Trichopeltis bicolor

XML Treatment for Trichopeltis doriae

XML Treatment for Trichopeltis feae

XML Treatment for Trichopeltis watsoni

XML Treatment for Trichopeltis kometis

XML Treatment for Trichopeltis latellai

XML Treatment for Trichopeltis cavernicola

XML Treatment for Trichopeltis muratovi

XML Treatment for Trichopeltis bellus

XML Treatment for Trichopeltis intricatus

XML Treatment for Trichopeltis reflexus

XML Treatment for Trichopeltis sutchariti
